# Depolarizing metrics for plant samples imaging

**DOI:** 10.1371/journal.pone.0213909

**Published:** 2019-03-14

**Authors:** Albert Van Eeckhout, Enric Garcia-Caurel, Teresa Garnatje, Mercè Durfort, Juan Carlos Escalera, Josep Vidal, José J. Gil, Juan Campos, Angel Lizana

**Affiliations:** 1 Grup d’Òptica, Physics Department, Universitat Autònoma de Barcelona, Bellaterra, Spain; 2 LPICM, CNRS, Ecole Polytechnique, Université Paris-Saclay, Palaiseau, France; 3 Botanical Institute of Barcelona (IBB, CSIC-ICUB), Barcelona, Spain; 4 Departament de Biologia Cel·lular, Fisiologia & Immunologia, Facultat de Biologia, Universitat de Barcelona, Barcelona, Spain; 5 Universidad de Zaragoza, Zaragoza, Spain; Università degli Studi di Milano, ITALY

## Abstract

Optical methods, as fluorescence microscopy or hyperspectral imaging, are commonly used for plants visualization and characterization. Another powerful collection of optical techniques is the so-called polarimetry, widely used to enhance image contrast in multiple applications. In the botanical applications framework, in spite of some works have already highlighted the depolarizing print that plant structures left on input polarized beams, the potential of polarimetric methods has not been properly exploited. In fact, among the few works dealing with polarization and plants, most of them study light scattered by plants using the Degree of Polarization (DoP) indicator. Other more powerful depolarization metrics are nowadays neglected. In this context, we highlight the potential of different depolarization metrics obtained using the Mueller matrix (MM) measurement: the Depolarization Index and the Indices of Polarimetric Purity. We perform a qualitative and quantitative comparison between DoP- and MM-based images by studying a particular plant, the Hedera maroccana. We show how Mueller-based metrics are generally more suitable in terms of contrast than DoP-based measurements. The potential of polarimetric measurements in the study of plants is highlighted in this work, suggesting they can be applied to the characterization of plants, plant taxonomy, water stress in plants, and other botanical studies.

## Introduction

Optical methods, as fluorescence microscopy or hyperspectral imaging, have proved their utility for the characterization and visualizations of plants and some of their structures [1–4]. One optical method, widely used for enhanced image contrast and characterization of samples are the polarimetric methods. However, they have barely been studied for the analysis of plants.

Polarization is a physical property of light exploited in a large number of applications, as a complementary tool to other techniques or constituting a completely different approach [5–7].

In recent decades, a large number of works have highlighted an interest in analyzing the polarimetric print left by biological samples when interacting with polarized light [8]. As a consequence, polarimetric techniques are commonly incorporated in different fields in order to study and characterize biological samples. For instance, polarimetric methods are successfully used in some medical applications, like in calculating the sugar concentration in blood in diabetics[9], or for the early diagnosis of some types of cancer [10, 11], including skin cancer [12, 13], colon cancer [14, 15], breast cancer [16], and others [17].

Polarized light is also used for curative processes [18–20]. Medical cases being treated with polarized light include severe second degree burns [18], wounds [19], leg ulcers, psoriasis and eczema [20], and the improvement of blood’s immunological response [21].

This well-known usefulness of polarized light and polarimetric techniques when dealing with biological tissues suggests their suitability in botanical applications. In the 80s, the interest in using polarized light for the characterization of botanical samples was explored by different authors [22–25]. In general, the studies were intended for remote sensing and were done to explore Degree of Polarization as an aid to vegetation classification [26]. They showed that light scattered at different leaf layers and structures presents different depolarizing characteristics and that this partially-polarized light may be described by the Stokes vector (see, for instance, [22, 27]).

After the above-mentioned pioneering works, most studies of plants based on polarimetric methods focused on the depolarization signal (as opposed to retardance or diattenuation) as it is the polarimetric channel leading to the most polarimetric sensitivity. Furthermore, nearly all works restrict their analyses to the use of the most basic depolarization metric, the Degree of Polarization (DoP) associated with the light reflected by or transmitted in plants [22, 25, 26, 28–30]. This DoP can be readily calculated from the Stokes vector parameters of the studied light [31, 32],

$$DoP=\frac{\sqrt{S_{1}^{2}+S_{2}^{2}+S_{3}^{2}}}{S_{0}},$$ (1)

where the Si (i = 0,1,2,3) are the Stokes parameters of the light transmitted, reflected, or scattered by the sample. Note that throughout this manuscript we use the Stokes-Mueller (S-M) formalism to describe the polarimetric characteristics of light and/or matter. The basic concepts of the S-M formalism are taken for granted in this manuscript and more details can be found in the specialized bibliography [31–34].

Some areas of interest related to botanical applications have been explored based on DoP calculations. For instance, the DoP has been applied to determine the water stress on plants [32], to monitor crop growth [29], or to discriminate land mines from natural backgrounds [35]. The measurement of polarization properties such linear, circular dichroism and birefringence as well as the DoP of light reflected by plants has been shown to be of great importance in research related to plant photosynthesis [36–38]. The effect of polarized and unpolarized light on the growth of some plants has also been investigated [39]. Vanderbilt et al. [30] found no evidence of hyperspectral variation in the polarized portion of the reflectance in the leaves of the five species they measured.

Despite the aforementioned collection of works based on the DoP indicator, polarimetric techniques have not been consolidated in botanical applications. Rather, in the last decade, they have fallen into oblivion, with the exception of some sporadic works [30, 36–39, 40].

The above-mentioned works show that different plant structures will depolarize light differently and thus, using depolarization as an observable, can be used to visualize structural properties (or changes) which remain veiled under nonpolarized light. Recent theoretical developments have shown that the depolarization phenomena can be more accurately described using a set of parameters deduced from the Mueller matrix than with the classical DoP deduced from the Stokes vector. The parameters obtained using the Mueller matrix have largely proved their significance in the evaluation of the depolarizing characteristics of samples, but they are not being applied in the botanical context. In fact, one of the purposes of this work is to reverse the decreasing tendency observed in recent years of botanical studies based on polarization and to show for the first time the potential of some depolarizing factors in plant imaging and characterization. We think that underscoring the significance of polarimetry in botanical applications may allow readers to adopt less destructive methods and to seek new botanical applications, which would have a high social impact, as plants are primary producers and the basis of the food chain.

In particular, we study different depolarization-related observables calculated using the Mueller matrix (MM) measurement in the botanical context. On one hand, we study the Depolarization Index *P*_*Δ*_ which was first proposed by J.J. Gil and E. Bernabeu [41], which constitutes a standard magnitude in the polarimetric community when dealing with depolarizers [31–34]. On the other hand, we focus on the so-called Indices of Polarimetric Purity (IPPs) [42, 43], which have been successfully used to enhance the image contrast of polarimetric images of animal tissues [44], unveiling physiologic structures which otherwise would have remained invisible. In fact, the interest in IPPs relies on the fact that each of them is sensitive to specific depolarization mechanisms. Since depolarization is related to the structure of tissues by the way they scatter light, the specificity of IPPs to different depolarization mechanisms can be used to finely discriminate among different tissue structures which scatter light in specific ways [44, 45]. The suitability of the aforementioned depolarizing factors and techniques is highlighted in this work by the study of light scattered by leaves of *Hedera maroccana*. At this point, we would like to emphasize that the choice of this particular species for this work was made because it was easily available to the authors. The choice is by no means exhaustive and shows that the use of polarized light can be extended to any type of leaf or vegetal tissue sample, provided it transmits enough light. The experimental measurements and polarimetric treatment discussed in this paper are provided to illustrate the suitability of the different depolarization indices in the study of vegetal tissues, which can be of interest in scientific and industrial areas related to, among others, pharmaceuticals, the food sector, and botany.

## Material and methods

In this section, we briefly review the mathematical fundamentals of different polarimetric indicators used to analyze the studied plants (subsection 2.1). We also include a brief description of the plant used for the polarimetric analysis, the *Hedera maroccana* (subsection 2.2), and we give some experimental details of the image polarimeter used to calculate the Mueller matrix of the samples (subsection 2.3).

### Mathematical background

We start first by reviewing the mathematical formulation of the depolarization metrics we use to characterize botanical samples. The depolarization index, *P*_*Δ*_, is a single-number metric that characterizes the depolarization of a MM and is defined as [41, 46],

$$P_{\Delta }=\frac{\sqrt{\sum_{ij=0}^{3}M_{ij}^{2}-M_{00}^{2}}}{\sqrt{3}M_{00}},$$ (2)

where *M*_*ij*_ are the different coefficients of the MM. The *P*_*Δ*_ equals 1 for nondepolarizing samples (samples that do not decrease the degree of polarization of any totally-polarized input beam) and equals 0 for an ideal depolarizer (a sample that fully depolarizes an input beam independently of its polarization). In fact, the *P*_*Δ*_ is proportional to the Euclidean distance between an ideal depolarizer and the specific depolarizer [42].

Thereafter, we review another set of depolarizing indicators that can also be obtained from the MM, three real magnitudes labelled as *P*_*1*_, *P*_*2*_, and *P*_*3*_ (with values from 0 to 1 each), known as Indices of Polarimetric Purity (IPPs) [42–44]. The idea behind IPPs is that the response of any depolarizer can be synthesized as the incoherent sum of four nondepolarizing components. In this context, IPPs represent the relative statistical weight of each one of the pure components, which allows us to differentiate between different types of depolarizers [45, 47]. Moreover, by using these three magnitudes as a coordinate system, a new representation of depolarizers, the so-called *Purity*-*Space*, is obtained. This is a very intuitive space because every possible depolarizer occupies a different spatial position in a tetrahedron inscribed within the *Purity*-*Space* [43, 47]. Thus, the physical interpretation of IPPs further synthesizes the depolarizing information of samples because every combination of IPPs is linked to a different depolarizing mechanism [45, 48] ― in contrast to the *P*_*Δ*_ indicator, which gives an overall depolarizing estimation.

These three IPP magnitudes are defined as follows in terms of the relative differences between the four eigenvalues (taken in decreasing order λ_0_≥λ_1_≥λ_2_≥λ_3_) of the covariance matrix H associated with the MM [43].

$$P_{1}\equiv \frac{\lambda_{0}-\lambda_{1}}{\mathrm{tr}H},P_{2}\equiv \frac{\lambda_{0}+\lambda_{1}-2\lambda_{2}}{\mathrm{tr}H},P_{3}\equiv \frac{\lambda_{0}+\lambda_{1}+\lambda_{2}-3\lambda_{3}}{\mathrm{tr}H}$$ (3)

Furthermore, the *P*_*Δ*_ can also be calculated from the IPPs as [43],

$$P_{\Delta }=\frac{1}{\sqrt{3}}\sqrt{2P_{1}^{2}+\frac{2}{3}P_{2}^{2}+\frac{1}{3}P_{3}^{2}}.$$ (4)

Nondepolarizing systems are characterized by *P*_*Δ*_ = *P*_*1*_ = *P*_*2*_ = *P*_*3*_ = 1. In the other limiting case, the values *P*_*Δ*_ = *P*_*1*_ = *P*_*2*_ = *P*_*3*_ = 0 correspond to an ideal depolarizer. In general, the indices of purity are restricted by the following inequalities [43],

$$0\leq P_{1}\leq P_{2}\leq P_{3}.$$ (5)

The aforementioned depolarizing metrics (Eqs (2) and (3)) are obtained from the MM of the sample and provide more complete and meaningful information of samples than basic DoP (Eq (1)).

### Plant sample description

In order to show the suitability of these MM-based metrics for experimental data, we have measured the Mueller matrix (MM) of a plant leaf. In particular, we measured a *Hedera maroccana* McAll. (Araliaceae) leaf, labelled as Sample A.

This is a climbing plant native to the Atlantic coast of Morocco. This species, which also grows in the Mediterranean area, is widely cultivated as an ornamental plant and is sometimes naturalized. The main diagnostic characteristics of this species are its foliar trichomes.

**Fig 1:**
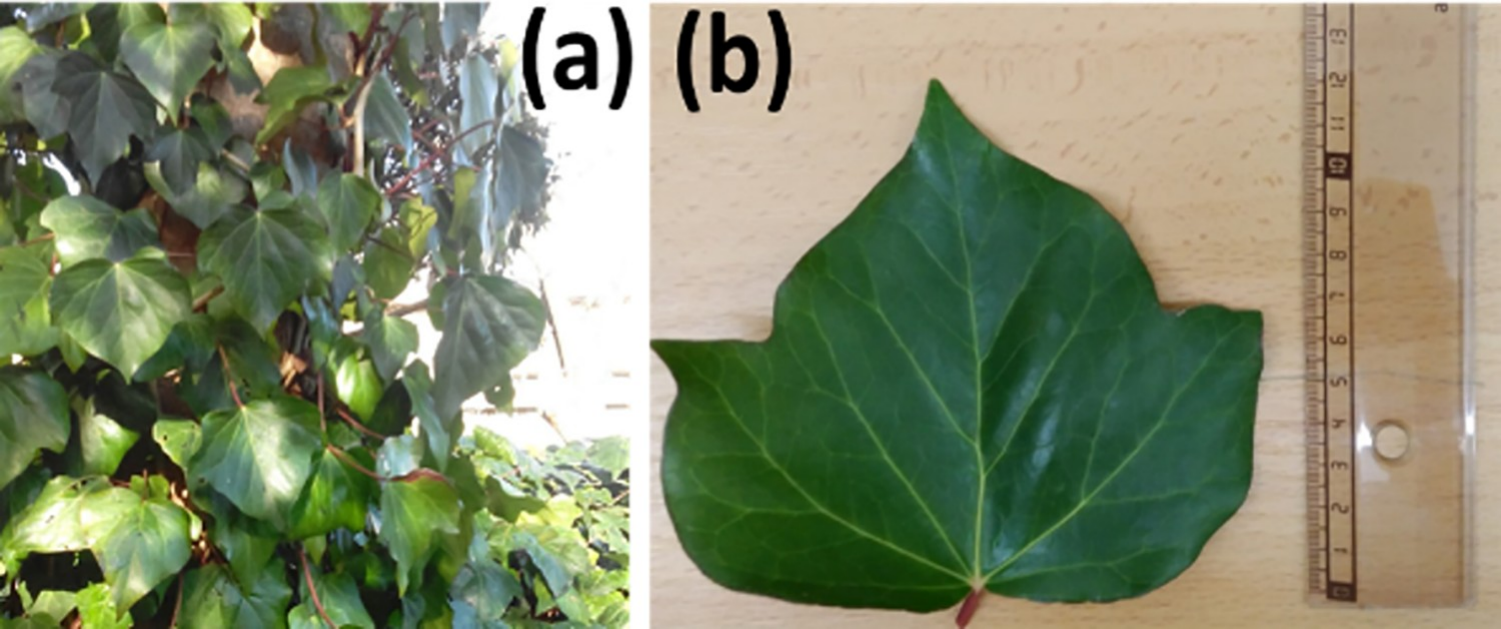
Plant sample used for the polarimetric analysis: (a) *Hedera maroccana* plant; (b) *Hedera maroccana* measured leaf (Sample A).

An herbarium voucher of the studied species is deposited in the Herbarium of the Botanical Institute of Barcelona (*Hedera maroccana*, BC843411). An image of the *Hedera maroccana* measured and considered in this work is given in Fig 1.

### Mueller matrix polarimeter

**Fig 2:**
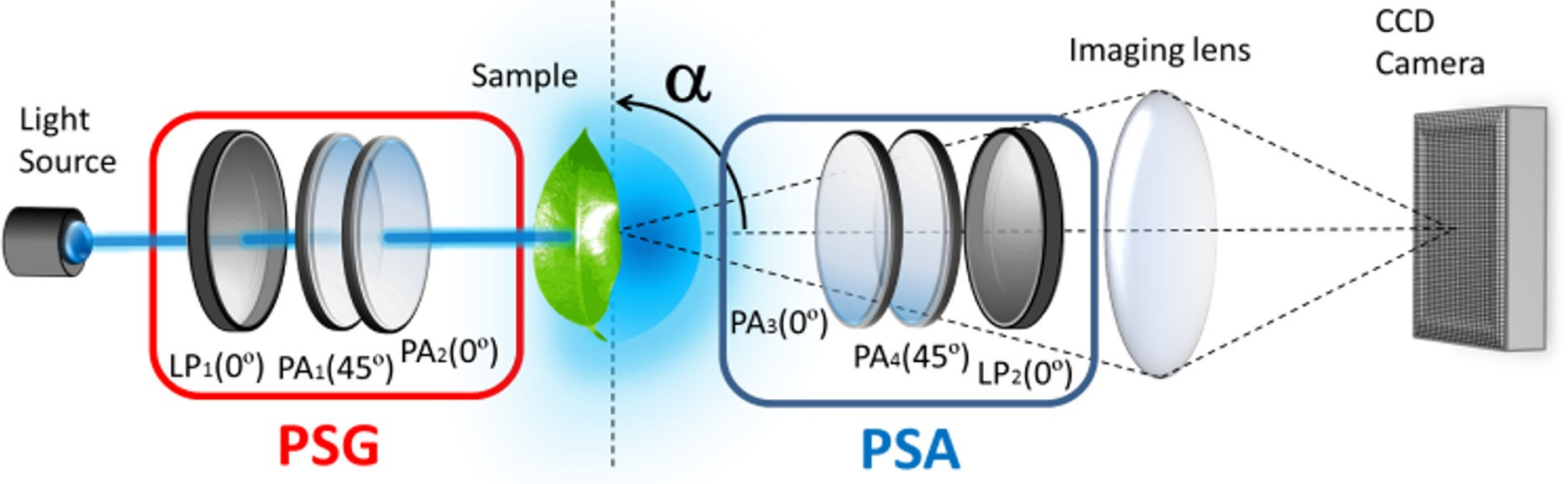
Scheme of the imaging polarimeter used to measure the Mueller matrices of plant leaves.

To determine the Mueller matrix (MM) of sample A, we used the complete image polarimeter sketched in Fig 2. The polarimeter mainly consists of two arms; the first one is used for illumination and polarization generation, while the second arm is used for imaging and polarization analysis. The sample is always placed between the two arms. Measures on sample A were always conducted in transmission configuration. What is more, the images shown in this work were conducted with the obverse of the leaf looking at the light source and the reverse looking at the CCD camera, as shown in the sketch given in Fig 2. It was also measured by flipping over the sample, i.e., with the reverse looking at light source and the obverse at the CCD camera. Results were quite similar, but the contrast obtained was slightly lower in this second case, so the first configuration was selected. Note that this is not a general result, and depending on the studied plant type, different contrast may be obtained by flipping over the sample. In fact, it will depend on the spatial distribution of the different leaf components and structures. Therefore, for each analyzed specimen, we recommend measuring both sides of leaves.

The first arm contains a light source and a Polarization State Generator (PSG) that allows for the controlling of the polarization of the light illuminating the studied sample. As a light source, we used the green channel (central wavelength of 530 nm and a FWHM of 10 nm, respectively), with a maximum output intensity of 1000 mA in both cases. To achieve a FWHM of 10 nm with the green channel, a dielectric bandwidth filter (by Thorlabs) was used. The PSG consists of a linear polarizer (LP1) oriented toward the laboratory vertical, followed by two Parallel Aligned (PA) liquid crystal panels. While the first liquid crystal panel, PA1, is placed at 45° to the laboratory vertical, the second one (PA2) is oriented at 0° to the vertical. By using this PSG scheme, and by properly addressing the voltages of the PA1 and PA2 elements, any fully-polarized state of polarization (SoP) can be generated [49]. This controlled illumination impinges a sample holder, where the botanical sample is set. Thereafter, light scattered by the sample is polarimetrically analyzed by using a Polarization State Analyzer (PSA). The PSA is composed of the same optical elements used in the PSG but is arranged in the reverse order. By using this PSA, any SoP can be measured [49].

Depending on the operation of the PSG and the PSA, the polarimeter can measure either Stokes vectors or Mueller matrices. If a single polarization state generated by the PSG is analyzed by the PSA, then the measurement corresponds to the Stokes vector representing the SoP of the beam scattered by the sample. The SoP described by this Stokes vector depends on both the initial polarization state created by the PSG and the optical response of the sample. From this SoP measurement, the corresponding DoP can be calculated according to Eq (1). On the other hand, if at least four well-different polarization states generated by the PSG and scattered by the sample are sequentially analyzed by the PSA, the collection of the resulting sixteen independent images can be used to compute the Mueller matrix of the sample [44, 50]. Note that a convergent lens images the sample plane to a CCD camera with a given magnification, so imaging polarimetry can also be performed. Moreover, the PSA system can be rotated from the specular direction (containing mainly non-scattered light) to an angle α (see Fig 2), which allows us to analyze the light scattered in different directions. A more complete discussion about the technical characteristics of the imaging polarimeter can be found in Ref. [50].

## Results and discussion

A discussion of the experimental results obtained is provided below. First, we treat the images of a leaf of *Hedera maroccana* (Sample A) by calculating the corresponding Degree of Polarization (DoP) of the forward scattering. Measures were conducted in transmission configuration (leaf transmittance of ~0.44% for 530 nm). Then, we provide a discussion interpreting the contrasts seen in the image in terms of the structures found in the leaf in a botanical framework (subsection 3.1). This interpretation serves as a benchmark (or gold standard) to be compared with the images obtained using the MM-based observables in order to show their potential in the analysis of plant structures (subsection 3.2).

### Plant samples contrast based on DoP measurements

**Fig 3:**
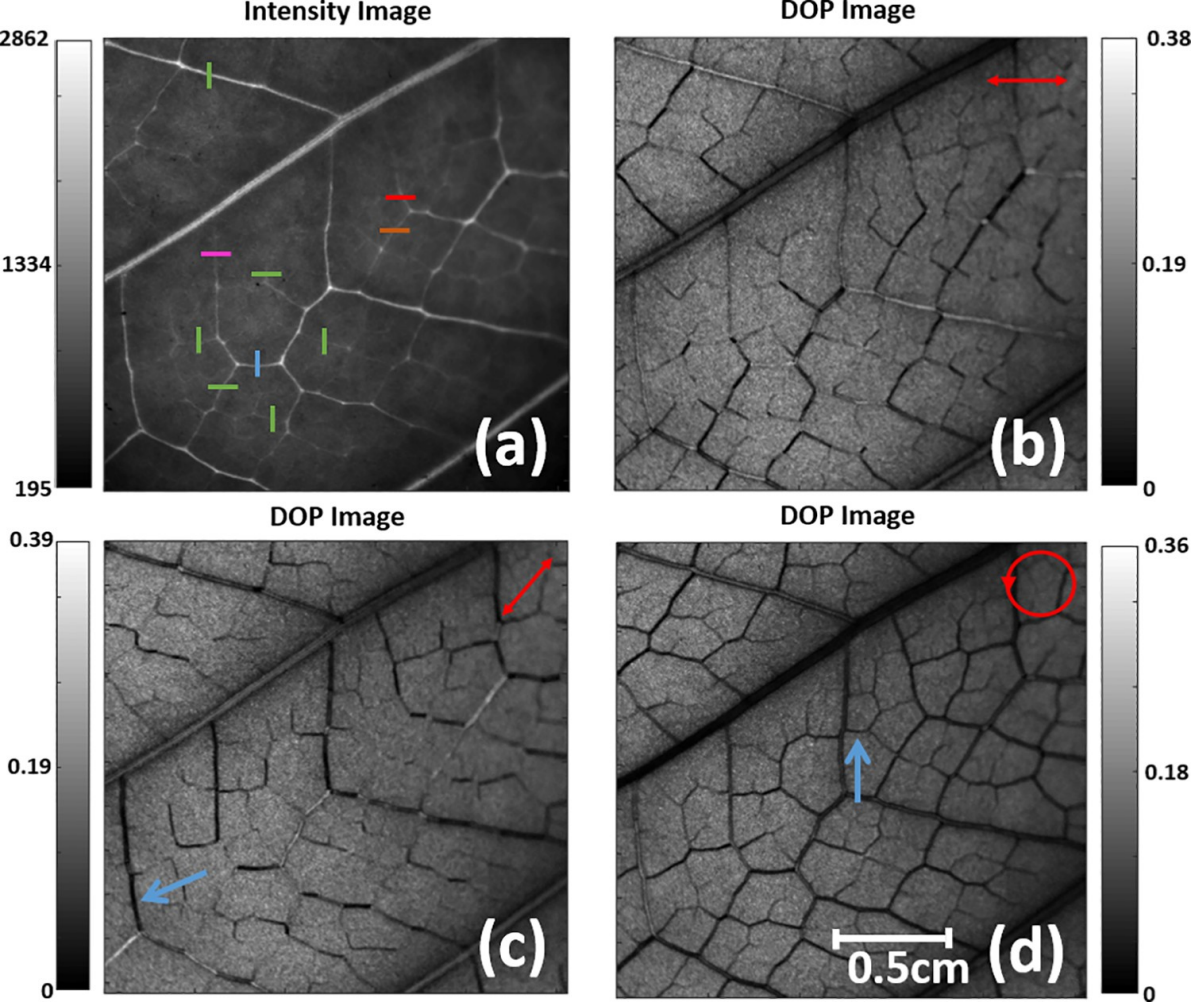
(a) Nonpolarized light intensity image of the Hedera maroccana (Sample A) obtained on transmission. (b)-(d) DoP image for an input polarization: (b) linear polarization at 0°; (c) linear polarization at 45°; (d) left-handed circular polarization. Input polarization is marked in red at the top-right corner of the images.

In this section we discuss the DoP images obtained from Stokes vectors scattered by sample A and measured in transmission configuration (α = 90° in Fig 2). Since the scattered SoPs depend on the initial polarization state, our discussion is based on a set of SoPs measured using the following incidents SoPs: linearly polarized in the horizontal direction (0LP), linearly polarized at 45° to the vertical direction (45LP), and, left-handed circularly-polarized (CP). The set of SoPs used in this work is arbitrary—they were chosen because they are linearly independent from each other and also intuitive. However, a different basis could have been chosen. The obtained images, shown in Fig 3, correspond to a Region of Interest (ROI) in the selected *Hedera maroccana* leaf (see Fig 1B) with dimensions of 1024x1024 pixels, which corresponds to an area of 2.2x2.2 cm on the leaf.

The image shown in Fig 3A corresponds to the coefficient m_00_ of the Mueller matrix of the sample, i.e. to the transmission of unpolarized light. The contrasts of this image reveal the presence of the primary (major) veins of the leaf, which constitute one of the more relevant structural features and which are even visible to the naked eye. By contrast, less visible in the intensity image but clearly defined in the DoP images (Fig 3B, 3C and 3D) are the secondary (smaller) veins. In fact, DoP images clearly stress the vascular bundles of highly basted and lignified walls.

It is important to notice that this improved contrast strongly depends on the selected input polarization, as the contrast among different secondary vein structures differs from one polarization to another. This fact is clearly observable by analyzing the visual information in Fig 3B (0LP), 3C (45LP), and 3D (CP). For instance, the leaf vein marked with a blue arrow in Fig 3C shows high contrast when using an input linear polarization at 45°, whereas almost no contrast is visible when using linear polarization at 0° (Fig 3B) or circular polarization (Fig 3D). When a linearly-polarized incident SoP is used, the contrast enhancement depends on the vein orientation in respect to the direction of the incident SoP. In contrast, the SoP orientation dependence is somewhat suppressed when a circular polarization is used (Fig 3D), with this polarization keeping an image contrast sufficient for the visualization of tiny veins.

**Fig 4:**
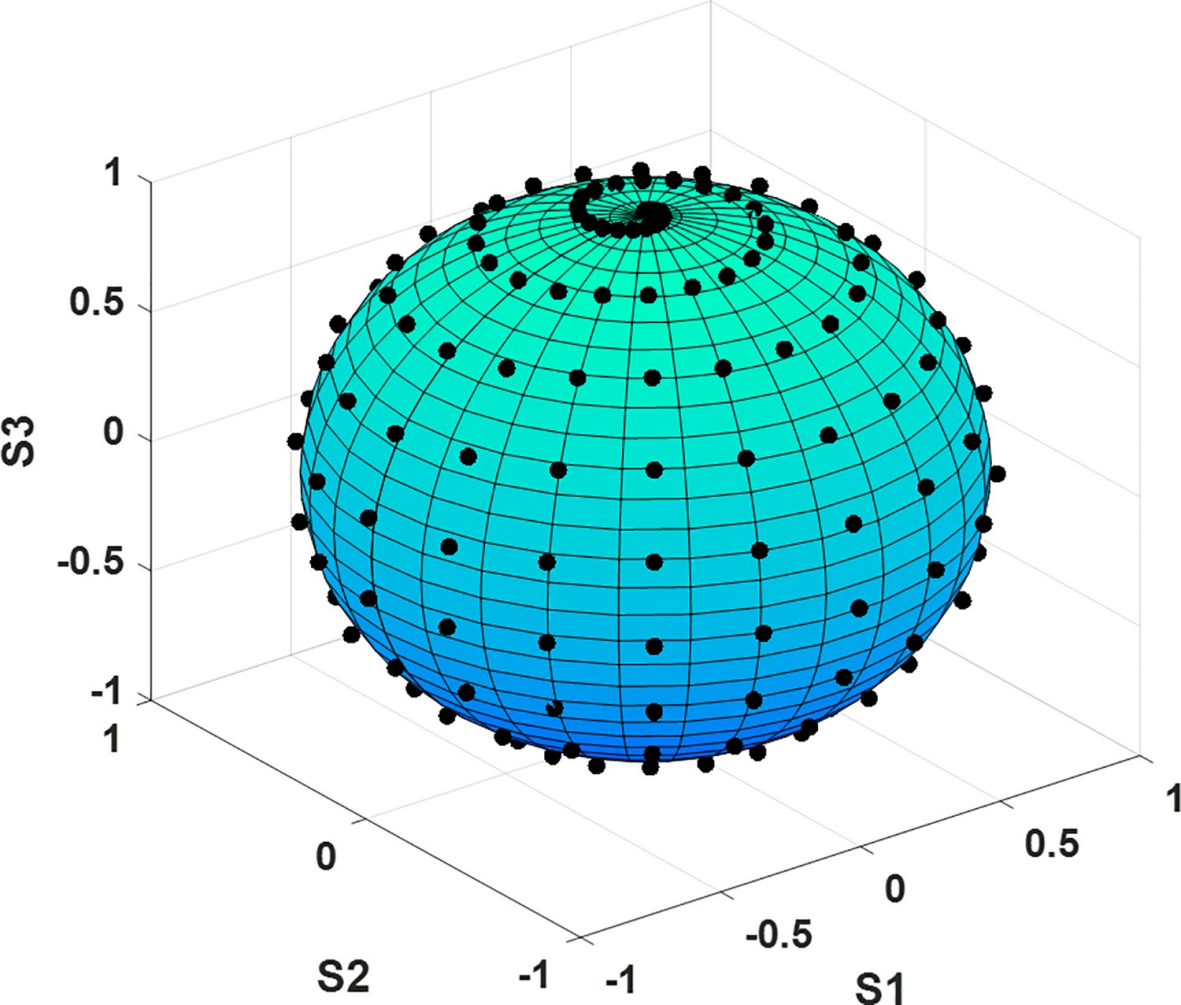
Poincaré sphere showing the location of the input polarizations (N = 200) used to study the dependence of the input polarization on the DoP-based images contrast.

This spatial dependence of the contrast of the leaf structures on the input polarization by using DoP-based images deserves special attention. In fact, the selection of the input polarization in botanical polarization-based studies is, in the majority of cases, arbitrary. Linear polarizations are most commonly used because of their simplicity to be generated (only a linear polarizer is required). To generalize the physical picture suggested by the images in Fig 3, we have measured the Mueller matrix for Sample A and we have analytically calculated the output polarization corresponding to a set of *N* input polarizations (according to well-known input-output Stokes linear relation scheme *S_output_* = *M_sample_*·*S_input_* [31, 32]). Afterwards, we calculated *N* DoP images corresponding to the *N* output Stokes array, according to Eq (1). To consider as widespread of a set of input polarizations as possible, we generated a collection of input polarizations equally distributed along the whole Poincaré sphere surface. In particular, the collection of input polarizations tested draw a spiral-like curve covering the whole Poincaré sphere surface (see Fig 4). They are described by the following parametric relation of the Stokes vector [51]:

$$\begin{array}{l} S_{k}={\left(\begin{array}{cccc} 1 & \cos2\theta_{k}\cos2\varepsilon_{k} & \sin2\theta_{k}\cos2\varepsilon_{k} & \sin2\varepsilon_{k} \end{array}\right)}^{T}\\ \{\begin{array}{c} \varepsilon_{k}=k\cdot \Delta \varepsilon -\frac{\pi }{4};\;\Delta \varepsilon =\frac{\pi }{2N_{\varepsilon }N_{\theta }};\\ \theta_{k}=k\cdot \Delta \theta ;\;\Delta \theta =\frac{\pi }{N_{\theta }};\\ k=1,\ldots ,N_{\varepsilon }N_{\theta } \end{array} \end{array}$$ (6)

where the *θ* and ε are the azimuth and ellipticity angles, describing the polarization ellipse [32]. Whereas *θ* goes from 0 to π, the angle ε goes from -π/4 to π/4 (from left to right handed, respectively). The parameter *N*_*θ*_ is the number of steps in each circle around the *S*_*3*_ axis and *Nε* is the number of circles around the *S*_*3*_ axis (see Fig 4).

In our particular calculation, we selected *N*_*θ*_ = 20 and *Nε* = 10, so a total number of N = 200 input polarizations are sampled, which are represented as black dots on the Poincaré sphere in Fig 4.

For the sake of visualization, the resulting collection of DoP images is arranged in video format included in the supplementary material accompanying this work (S1 Video). In fact, S1 Video consists of 200 frames where each frame shows the DoP image of Sample A calculated for a different input polarization. By varying the input polarization, we see how the contrast in different veins is modified (or even appears and disappears), and thus, S1 Video constitutes clear evidence of the high dependency of each particular leaf structure contrast on the input polarization. This dependence can be explained by the fact that veins and other structures are made of highly oriented polymers that present certain anisotropic (birefringent or dichroic) response. The components that mainly provide birefringent response in plants (as well as dichroism when some pigments are present) are the cellulose fibrils, and in particular, the microcrystals of which cellulose fibrils are made of [52–55]. These microcrystals tend to be oriented in the direction of the large structures. Oriented polymers generate a form of anisotropy (linear or circular, depending on which oriented polymer is considered) which implies that they do not isotropically scatter light in all directions of the space. This fact can explain the contrast dependence on the vein direction observed in DoP images when the illumination was linearly polarized. In contrast, since the electric field of a circular polarization vibrates with the same probability in all directions perpendicular to the propagation direction, the orientation dependence in DoP images obtained with circularly-polarized illumination is less evident. However, the maximum contrast obtained with circular polarization is half the maximum contrast obtained with linearly-polarized light.

**Fig 5:**
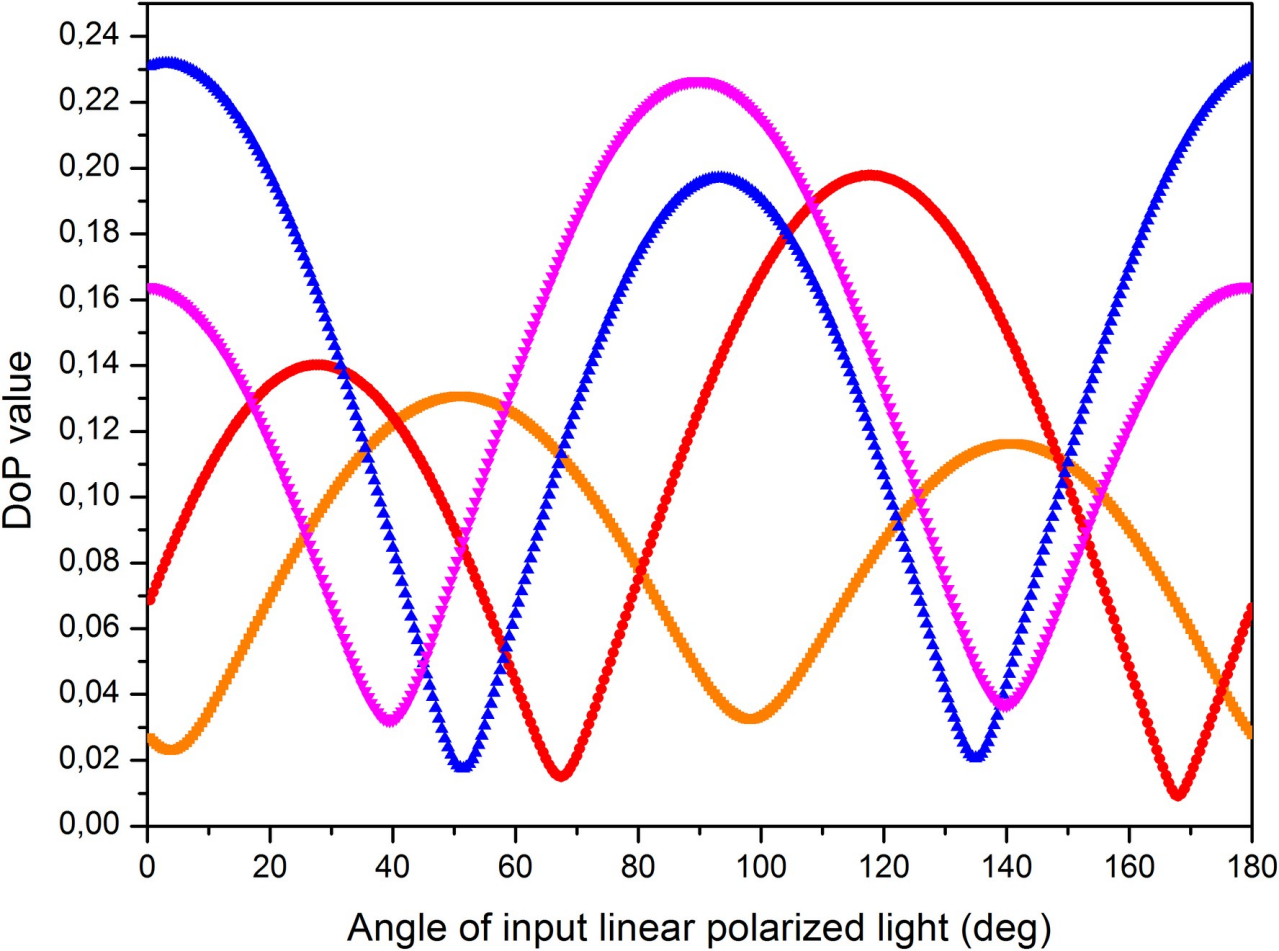
DoP values as a function of the input linear polarization orientations. Orange (squared), red (circle), blue (triangle) and purple (inverted triangle) curves correspond to the segments of the same color in Fig 3A.

As above-stated, we observe a clear relation between the visualization of the veins in the *Hedera marroccana* leaf and the input polarization. We further investigated this fact by analyzing the correlation between the DoP values of veins with different orientations and the input polarization orientation. To this aim, we calculated different *Hedera marroccana* DoP images corresponding to a set of different linear input polarizations, which were calculated by setting *ε*_*k*_ = 0 and *N*_*θ*_ = 200 in Eq (6) (i.e., the 200 equispaced linear polarizations placed over the Poincaré sphere equator were evaluated). From the different DoP-based images, we calculated the averaged value of the DoP obtained at three consecutive pixels in a segment over four different veins with different orientations (see orange, red, blue and purple segments in Fig 3A). The obtained results are represented in Fig 5.

Data in Fig 5 reveals a strong dependence between the DoP values and the orientation of the input linear polarization, following an approximately sinusoidal relation. All the analyzed veins (colored segments in Fig 3A) follow the same tendency, but the positions for the DoP maximums (minimum depolarization) and minimums (maxima depolarization) are related to different orientations of the input linear polarization (i.e., there are horizontal shifts between DoP curves obtained for veins with different orientations). In fact, we observed that the orientation of the input linear polarization for which a maximum value of the DoP is measured, it is parallel (coincides) with the orientation of the vein in the leaf. This situation has sense because as commented before, veins are made of highly oriented vascular bundles (oriented organic polymers). When linear polarization is oriented parallel to the leaf veins, and for symmetry reasons, it is also likely to be oriented parallel to the global dipole of the oriented molecules from with the vein is made. Although the measurements presented in this work are done in relatively transparent spectral region, the interaction of light with matter is always present and higher when the polarization is parallel to the dipoles (from which the matter is composed) than when the polarization is perpendicular to them. So, it can be said that light will be more efficiently absorbed when it vibrates parallel to the molecules than otherwise. If absorption is enhanced, then, the amount of scattered light respect to direct light decreases because the optical path of scattered light is longer than that of direct light. Let us recall that depolarization arises because there is an incoherent superposition of direct and scattered light contributions when it is detected by the CCD camera. If due to the above mentioned reasons the detected component related to direct light dominates, then the DoP increases (light polarization becomes purer). When light is polarized perpendicularly to the direction of molecular dipoles, the interaction of light with them (and thus, absorption and subsequent re-emission of scattered light), is also minimized. Again, in this situation, but for different reasons, the ratio between direct (non-scattered) to scattered light is favorable to the direct light component reaching the detector, which leads to an increase of the measured DoP. When light is neither parallel nor perpendicular to the material dipoles, the ratio of scattered to direct light reaching the detector increases thus leading to a decrease of the DoP. For symmetry reasons, light polarized at 45° with respect to the material dipoles represents a particular case for which the DoP reaches a minimum value (maxima depolarization).

To summarize this correlation between the vein angular orientation in the leaf and the orientation of the input linear polarization providing the maximum DoP value, these two quantities are provided in Table 1 for the four veins studied.”

**Table 1:** Correlations between the vein orientation and the maximum DoP value for different veins in the *Hedera maroccana* leaf (orange, red, blue and purple segments in Fig 3A).

Segments	Vein orientation with respect to the lab horizontal (deg)	Input linear orientation (in deg) for the maximum DoP
**Orange**	51±1	51±0.5
**Red**	119±1	117.5±0.5
**Blue**	3±1	3±0.5
**Purple**	89±1	90±0.5

The above-stated dependence of the *Hedera maroccana* contrast with input polarization was further studied from a more quantitative point of view. To do so, the visibility V can be defined as a function of the input polarization particular index (k parameter in Eq (6)). Note that the visibility can be calculated for any arbitrary point on the image. In our case, we focused on two particular secondary veins, which are oriented at 90° degrees one to each other (visibility of the orange and blue segments in Fig 3A). These visibility values were obtained according to the following equation,

$$V=\frac{I_{\max}-I_{\min}}{I_{\max}+I_{\min}},$$ (7)

where Imax and Imin stand for the maxima and the minima intensity of the selected segments.

**Fig 6:**
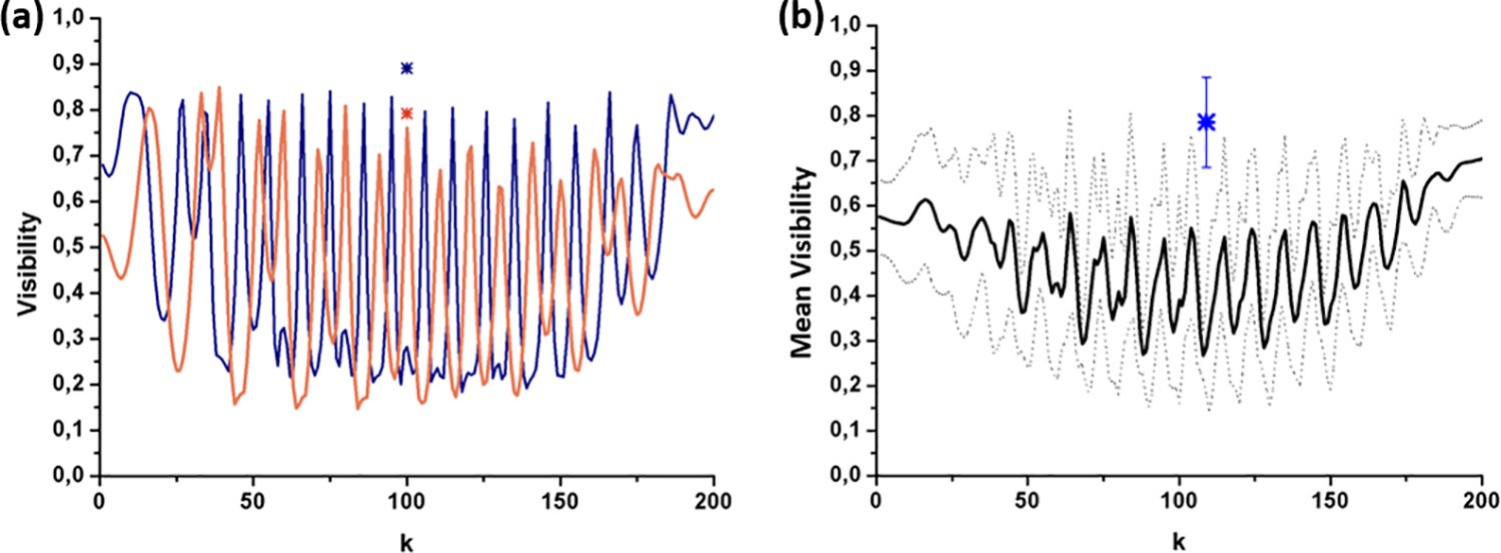
Visibility values calculated from DoP images corresponding to 200 different input polarizations (k parameter). (a) Orange curve and blue curve provide the visibility related to the orange and blue segments in Fig 3A, respectively; and (b) Mean visibility (black curve) as a function of the input polarization, calculated from 10 different segments (orange, blue and 8 green lines in Fig 3A) arbitrarily selected along the leaf. The corresponding standard deviations values are given by the upper and lower dashed black lines.

The results are shown in Fig 6A, where we see the visibility value for each tested input polarization (i.e., as a function of the input polarization index k in Eq (6); ranging from 1 to 200). The orange and blue curves in Fig 6 provide the visibility values as a function of k for the orange and blue segments in Fig 3A, respectively. They reveal that the significant dependence of the image contrast as a function of the three input polarizations discussed in Fig 3 (linear polarizations at 0° and 45° and right-handed circular polarization) is generalized for all the mapped input polarizations, as provided by the high variation of the visibility observed in Fig 6A both for the orange and blue curves (with peak-to-valley visibility variations from approximately 0.15 to 0.85 in both cases). It is also clear that the maximum visibility for different structures in the leaf (orange and blue segments in Fig 3A) are obtained for different input polarizations, as shown by the peaks displacement observed between the orange and blue curves. Therefore, we confirm that the visibility of a particular plant structure depends on the input polarization (high visibility variations with the *k* parameter in Fig 6A). What is more, we also prove that different plant structures present different visibility responses to the input polarization (as shown by the different curves in Fig 6A).

For the sake of generalization, the same study was repeated using a larger number of structures. In particular, the visibility of 10 different segments arbitrarily chosen all along the whole image was calculated (see orange, blue and 8 green segments in Fig 3A). The average visibility as a function of the input polarization index k is represented as a black curve in Fig 6B. Note that for each value of k (x-axis), the value of the mean visibility is calculated in the following way: the visibility for each one of the 10 random segments is calculated, then the mean value of these 10 visibilities is obtained, and this is the value represented in Fig 6B. In addition, the corresponding upper and lower deviations, included as dashed lines, were calculated from the standard deviation.

We see that by considering different plant structures at the same time (10 segments), the corresponding mean visibility (black line) is considerably reduced. Note that if some input polarization (k value) were capable of obtaining high visibility values for the 10 plant structures at the same time, some point of the visibility black curve would be close to 1. However, we see that all the mean visibility values are lower than 0.7, with the majority of them restricted to values lower than 0.6 for all the input polarizations. This result generalizes the discussion related to Fig 6A, and confirms that a particular input polarization provides very different visibility values for different plant structures. The same idea is observed in the large standard deviations associated to the mean visibility values (dashed lines in Fig 6B).

In summary, the images in Figs 3 and 6 and S1 Video demonstrate that the contrast of DoP-based images is highly dependent on the input polarization, so an optimal selection of the input polarization is a crucial issue. What is more, the visibility of different spatial structures of the plant show a large variation (Fig 6B) when a particular illumination polarization is chosen. Therefore, the optimum contrast related to each specific biological structure is obtained by selecting different input polarizations. Considering the vast majority of polarimetric methods conducted on plants so far are based on DoP measurements and using a particular input polarization (usually linearly-polarized light), the above-provided study reveals that those methods never provide the best possible contrast simultaneously for all the biological structures present in the plant. Thus, the use of new techniques to better enhance the overall contrast of polarized images of plants is required.

### Contrast of plant samples based on MM metrics: P_Δ_ and IPP indicators

**Fig 7:**
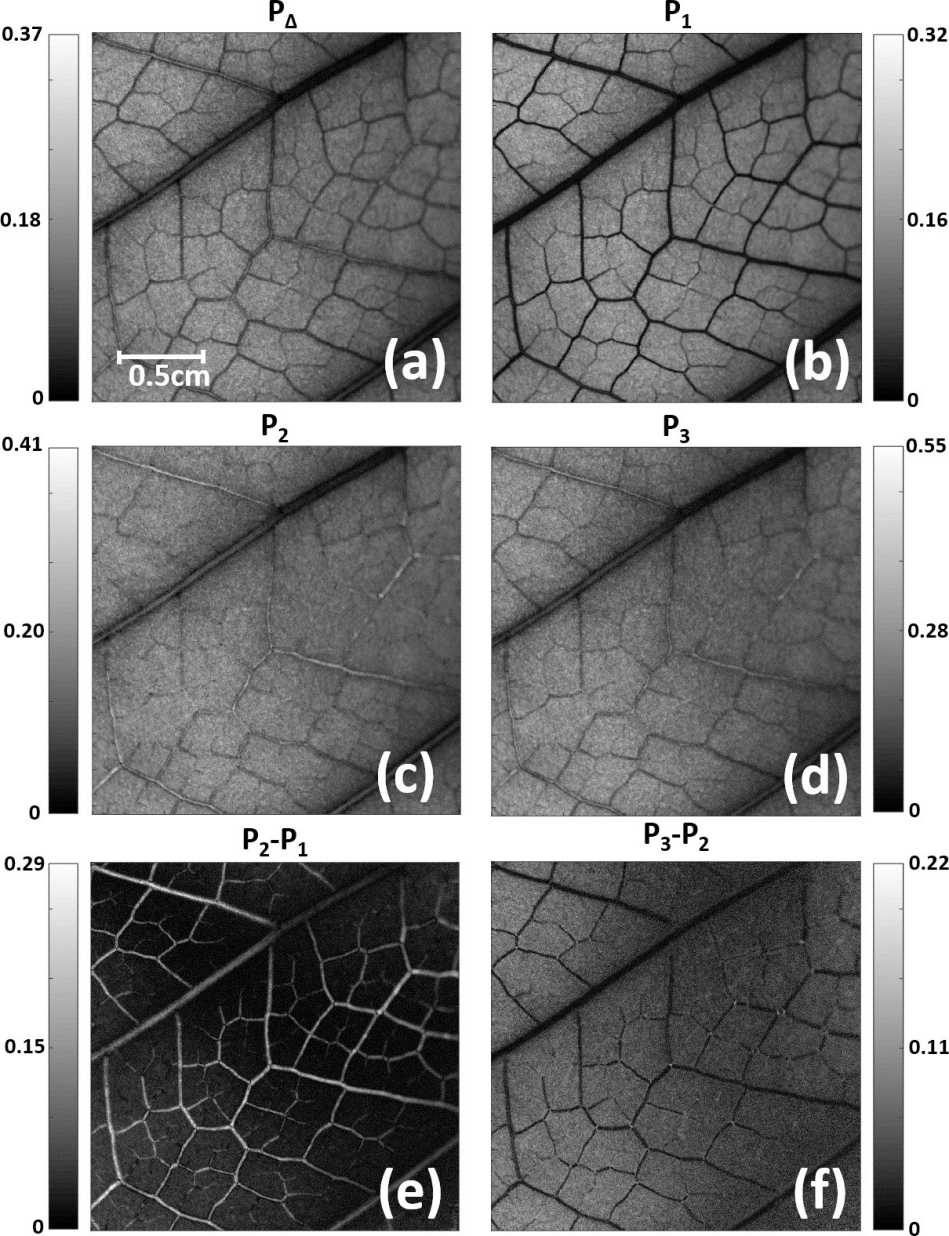
*Hedera maroccana* (Sample A) images obtained by using different depolarizing based indicators: (a) Depolarization Index *P*_*Δ*_; (b)-(d) Indices of Polarimetric Purity (IPPs), *P*_*1*_, *P*_*2*_ and *P*_*3*_. Sample A images obtained by combining different IPP channels: (e) *P*_*2*_-*P*_*1*_; and (f) *P*_*3*_-*P*_2_.

In the present section, we discuss the results obtained when MM-based observables are used and we compare them with the results obtained for DoP-based images. In particular, the depolarization metrics reviewed in section 2.1 were calculated for Sample A from the experimental MM of the sample. For comparison with images in Fig 3 (DoP-based images), Sample A images for the *P*_*Δ*_, *P*_*1*_, *P*_*2*_ and *P*_*3*_ polarimetric purity indices are given in Fig 7A–7D, respectively. We see that different polarimetric channels provide different contrast visualization of the plant structures. This can be understood by taking into account the physical interpretation of these metrics. Whereas *P*_*Δ*_ gives a measure of an overall depolarization capability of the sample [41], i.e. it depolarizes more or less (from 0 to 1), the IPPs are related to the inherent depolarizing mechanisms of samples, and thus can differentiate among different kinds of depolarizers [42, 43, 45]. From all the obtained results, the best image contrast is achieved for the *P*_*1*_ channel (Fig 7B), clearly showing the vascular bundles of highly basted and lignified walls constituting the veins in the *Hedera maroccana* sample. This contrasted visualization of the veins indicates that they scatter light in a very different way than other structures in the plant. More precisely, the veins in Sample A can be understood as equivalent depolarizers consisting of an incoherent addition of two nondepolarizing Mueller matrices [31, 32]. Note that this analysis is correct for the studied particular case of Sample A, but the best-contrasted images for other plants could be obtained with *P*_*2*_ or *P*_*3*_ channels if different inherent depolarizing mechanisms were predominant.

In this scenario, the study of IPPs channels is highly recommended because they synthesize and magnify the overall depolarizing information given by DoP images (section 3.1), leading to higher contrast.

By comparing the results in Fig 3 (DoP-based images) with those in Fig 7 (MM-based images), we realize that a given input polarization can enhance the polarimetric response of a particular structure of a plant with this polarization maximizing the depolarizing response of this particular biological structure. However, when different depolarizing mechanism origins (plant scatters with different densities, concentrations, organizations, sizes, etc.) are at different spatial locations, as is the usual case of a biological image, a particular polarization illumination does not reveal all the properties of the plant (check the dependence of the spatial image contrast on the input polarization in Figs 3 and 6 and S1 Video). Unlike this, by calculating the MM of the plant, the full polarimetric information is encoded in the matrix, as MMs describe the polarimetric behavior in polarimetric samples [31–34]. In this scenario, a proper decoding of the polarimetric information can reveal all characteristics of the sample. In such a situation, the analysis of a set of different depolarization metrics based on the MM arises as a promising strategy, as they provide an overall visualization of scattering structures in the plant. For instance, we have shown how, in the particular case of *Hedera maroccana*, the *P*_*1*_ channel (Fig 7B) is clearly better than any DoP image obtained using any other input polarization state (Fig 3A–3D).

Although we review here the particular case of Sample A, we have also studied other plant taxa (*Spathiphyllum* sp., *Hibiscus syriacus* L., *Prunus dulcis* (Mill.) D.A.Webb, *Arum italicum* Mill.). In all these cases, MM metrics provided an overall image contrast enhancement when compared with standard DoP-based measurements. In particular, the *P*_*1*_ channel tends to provide the highest contrast in the majority of studied cases.

Some authors have also pointed out that the combination of different IPP channels may lead to a visualization improvement [44, 47]. For instance, in the particular case of Sample A (Fig 7), we see how, as stated before, the *P*_*1*_ channel provides a significant contrast of the plant veins, whereas these structures are more poorly contrasted in the *P*_*2*_ channel (*P*_*2*_ image shows quite a constant spatial intensity with blurred vein structures). Therefore, the direct differences between *P*_*2*_-*P*_*1*_ channels could be understood as the removing of certain image background, which leads to a possible image enhancement for some plant structures. This hypothesis is compatible with the structure of polarimetric randomness [56] given by the *characteristic* (or *trivial*) *decomposition* [57], whose coefficients are precisely the differences *P_i_*−*P*_*i*−1_. To test this situation, we have also calculated the *P*_*2*_-*P*_*1*_ (direct difference between images in Fig 7C and 7B) and *P*_*3*_-*P*_*2*_ (direct difference between images in Fig 7D and 7C) images for the Sample A, and the corresponding results are given in Fig 7E and 7F, respectively. We see well-contrasted images in both cases, especially for the *P*_*2*_-*P*_*1*_ channel (Fig 7E), leading to the best contrast of the primary and secondary veins in Sample A.

To highlight this image contrast enhancement provided by Mueller matrix-based metrics from a quantitative point of view, we have examined the visibility of the orange and blue pixel-segments studied in Fig 3. Let us now turn to the MM-based images in Fig 7. As a reminder, the two orthogonal segments set in Fig 3 are plotted again in Fig 7B. In particular, the visibility values corresponding to the direct channels P_Δ_, P_1_, P_2_, P_3_, as well as for the combined channels P_2_-P_1_ and P_3_-P_2_, were calculated according to Eq (7), and both for the orange and blue segments (i.e., we tested two different secondary veins in Sample A). The results obtained are summarized in Table 2, where we observe how the P_1_ and P_2_-P_1_ channels are those providing the best visibility values for both the orange and blue segments. This result was expected because the P_1_ and P_2_-P_1_ polarimetric images of the *Hedera maroccana* leaf provided the best visualization for Sample A structures (see Fig 7).

**Table 2:** Visibility values *V* for different polarimetric indicators (different columns) corresponding to the orange and blue segments in Fig 3A.

Segment	m_00_	P_Δ_	P_1_	P_2_	P_3_	P_2_-P_1_	P_3_-P_2_
**Orange**	0.1	0.34	0.71	0.16	0.18	0.89	0.54
**Blue**	0.28	0.26	0.68	0.25	0.19	0.79	0.65

For the sake of comparison with the DoP-based images, the visibility values obtained for the *P*_*2*_-*P*_*1*_ images (i.e., the largest visibility values in Table 2) are represented in Fig 6A as an asterisk (an orange and blue asterisk for the orange and blue segments in Fig 7B, respectively). We want to note that the visibility calculated for the *P*_*2*_-*P*_*1*_ channel (asterisks in Fig 6A), or for any other MM-based metric, does not depend on the input polarization because they are calculated from the MM of the sample (see metrics in Section 2.1). In fact, the Mueller matrix can be understood as the polarimetric transfer function of the system, linearly relating the input and output polarizations [31–34], and only depends on the polarimetric characteristics of the sample.

In the case of the vein in Sample A, highlighted in the orange segment, the visibility value for the *P*_*2*_-*P*_*1*_ channel is equal to 0.79 (orange asterisk in Fig 6A), with this value being very close to the maximum visibility value obtained from DoP-based images (0.85 for the value k = 39 in the orange curve in Fig 6A). On the other hand, in the case of the blue segment, the visibility value for the *P*_*2*_-*P*_*1*_ channel is equal to 0.89 (blue asterisk in Fig 6A), with this visibility being larger than any other visibility obtained from DoP images (the largest DoP-based visibility for the blue curve is 0.84, obtained for the polarization index value k = 166).

Therefore, unlike DoP-based methods, by using the MM-based depolarizing metrics we obtain a reasonably good visibility for the two studied segments simultaneously (more than 0.78), and without any dependence on the input polarization. In particular, despite the fact that some specific input polarization (which must be found using some optimization method) may lead to the largest visibility for a specific structure of the plant (e.g., orange segment visibility of 0.85 for the input polarization k = 39; see Fig 6A), this same input polarization will degrade the visibility of other structures in the plant (blue curve for the same k, visibility value of 0.26). Therefore, the fact that using *P*_*Δ*_ and IPP indicators for the full image of the plant provides a nice overall contrast without the necessity of optimizing the input polarization proves this is a more adequate approach for the characterization of plants through polarizing images.

Finally, the adequacy of MM-based metrics in the visualization of different plant structures is further highlighted by generalizing the above-described visibility study to 10 different pixel-segments arbitrarily selected along the plant (the same 10 segments shown in Fig 3A that were previously used to test veins placed at different spatial positions on the leaf, data in Fig 6B). In particular, we calculated the mean visibility (average of the visibility values for the 10 segments) corresponding to the *P*_*2*_-*P*_*1*_ channel. The corresponding standard deviation was also calculated. To illustrate the comparison with the DoP-based approach, the calculated mean visibility is marked with a blue asterisk in Fig 6B along with its corresponding error bar. We observe how the mean visibility obtained from the *P*_*2*_-*P*_*1*_ channel is significantly higher (0.77, blue asterisk in Fig 6B) than the mean visibility calculated using DoP images (black curve in Fig 6B), independently of the input polarization (k parameter). This result highlights the suitability of the MM-based depolarizing metrics for plant imaging.

## Conclusion

In this work we presented the benefits of polarimetric methods for the inspection of plants. Although polarimetric methods have widely proved their suitability in biological applications, for instance in medical applications, they have not been extensively exploited in botanical applications. In particular, despite the fact some authors have studied different plants using polarized light, the number of works in this topic is not very extensive, and those that do exist mainly focus on the study of the Degree of Polarization of light dispersed by plant samples.

However, methods for polarimetric analysis of data have been largely improved in recent years. We proved how current polarimetric tools, based on the calculus of the Mueller matrix of the samples, can be beneficial in extracting information about plant structure. In fact, polarimetric tools provide images showing a larger contrast in some plant structures (or even show structures hidden in the intensity images) than nonpolarized intensity images. Furthermore, they have proven to be more suitable than polarimetric approaches based on the Degree of Polarization evaluated from the Stokes vector of scattered light.

A qualitative/quantitative polarimetric analysis of a *Hedera maroccana* leaf is provided in this work. The contrast of some leaf structures which are hidden in nonpolarized light intensity images (such as secondary veins), can be revealed by DoP images. However, we proved that such structures, like veins with different spatial orientation, present very different visibility values as a function of the input polarization. As a consequence, no input polarization is able to provide high visualization of all structures at the same time. In contrast, we proved how some polarimetric indicators evaluated using the Mueller matrix provide a much better overall visualization of plant structures and are highly recommended over DoP-based images. In particular, the depolarization index, *P*_*Δ*_, and the Indices of Polarimetric Purity, IPPs, were used to study the *Hedera maroccana*. Among these indices, we have shown that both *P*_*1*_ and *P*_*2*_-*P*_*1*_ channels provide the best contrast of the principal and secondary vein systems of the leaf. Analyses conducted on sample A were repeated on different *Hedera maroccana* leaves (sampling of 5 leaves), obtaining analogous results.

The examples provided in this work prove that polarimetric methods can be successfully used in botanical applications and the methods described could be of interest in a wide number of botanical applications. For instance, cell membrane depolarization potential can be a transient situation due to different factors: biotic elicitor for phytoalexin production in vitro culture, effect of feeding on plant leaf, interaction between root plant and Rhizobium bacteria, etc. The analysis of polarimetric imaging of plant tissues is then a useful parameter in order to verify the membrane integrity and function. The methods could also be applied in diverse botanical areas, as for instance, in plants characterization of structures and plant taxonomy, evolution of plant specimen, hydric stress determination, and for early detection of some plant diseases.

## Supporting information

S1 Video Video consist of 200 frames where each frame shows the DoP image of *Hedera maroccana* leaf calculated for a different input polarization. (AVI)
